# Supplementary material for: Complexome profiling of the Chlamydomonas psb28 mutant reveals TEF5 as an early PSII assembly factor
Source: Plant Cell. 2025 Mar 17;37(6):koaf055. doi: 10.1093/plcell/koaf055 (PMC12164590; doi:10.1093/plcell/koaf055)
Supplement: koaf055_Supplementary_Data [file koaf055_supplementary_data.zip › psb28_interactive.zip!/plots/intensity/qualityPlots/peptides/TEF21_Cre09.g388750.t1.2.html]

### Cre09.g388750.t1.2

Trivial name: TEF21  
SeqCoverage [median]: 22.20  
Mass: 67.91 kDa  
  
 

| Sequence | MissedCleavage | Mass [Da] | wtIntensity | muIntensity | Score |
| --- | --- | --- | --- | --- | --- |
| AAYGDVVAINLINHK | 0 | 1596.86 | 0.00 | 1257.17 | 39.42 |
| AQVLTEPGADKNSANR | 1 | 1669.84 | 0.00 | 2614.21 | 76.15 |
| IADRDADPVAAAAPLASPQR | 1 | 2004.04 | 0.00 | 2694.84 | 72.77 |
| IAPDVNR | 0 | 783.42 | 0.00 | 1220.84 | 86.51 |
| QLPGLHFAGGK | 0 | 1123.61 | 0.00 | 1078.85 | 72.20 |
| QVAALGPNSSAVYELVR | 0 | 1772.94 | 0.00 | 1522.63 | 120.87 |
| SATLTLIAR | 0 | 944.57 | 0.00 | 2594.06 | 103.83 |
| TFGGVIDDGVK | 0 | 1106.56 | 0.00 | 462.76 | 61.65 |
| TFGGVIDDGVKAVTR | 1 | 1533.82 | 0.00 | 1005.06 | 30.48 |
|  |  |  | 0.0 | 14450.4 |  |
